# Generating retinal flow maps from structural optical coherence tomography with artificial intelligence

**DOI:** 10.1038/s41598-019-42042-y

**Published:** 2019-04-05

**Authors:** Cecilia S. Lee, Ariel J. Tyring, Yue Wu, Sa Xiao, Ariel S. Rokem, Nicolaas P. DeRuyter, Qinqin Zhang, Adnan Tufail, Ruikang K. Wang, Aaron Y. Lee

**Affiliations:** 1 Department of Ophthalmology, University of Washington, Seattle, WA USA; 2 eScience Institute, University of Washington, Seattle, WA USA; 3 Department of Bioengineering, University of Washington, Seattle, WA USA; 4 Moorfields Eye Hospital NHS Foundation Trust, London, UK

## Abstract

Despite advances in artificial intelligence (AI), its application in medical imaging has been burdened and limited by expert-generated labels. We used images from optical coherence tomography angiography (OCTA), a relatively new imaging modality that measures retinal blood flow, to train an AI algorithm to generate flow maps from standard optical coherence tomography (OCT) images, exceeding the ability and bypassing the need for expert labeling. Deep learning was able to infer flow from single structural OCT images with similar fidelity to OCTA and significantly better than expert clinicians (P < 0.00001). Our model allows generating flow maps from large volumes of previously collected OCT data in existing clinical trials and clinical practice. This finding demonstrates a novel application of AI to medical imaging, whereby subtle regularities between different modalities are used to image the same body part and AI is used to generate detailed inferences of tissue function from structure imaging.

## Introduction

Optical coherence tomography (OCT) is a non-invasive imaging modality of structural retina *in vivo*. Since its development in 1991, OCT has become essential in diagnosing and assessing most vision-threatening conditions in ophthalmology^1^. A recent advance in OCT technology led to its counterpart, OCT angiography (OCTA), which measures blood flow in retinal microvasculature by obtaining repeated measurements of phase and intensity at the same scanning position^2,3^. While OCTA can theoretically be obtained using the same OCT hardware, in practice, OCTA requires both hardware and software modifications to existing OCT machines. OCTA can visualize both superficial and deep capillary plexus of the retinal vasculature without an exogenous dye, unlike fluorescein angiography, enabling better detection of overall retinal flow without potential side effects^4^. Despite the advantages, the use of OCTA is not as widespread as OCT, due to its cost and limited field of view (FOV) on currently commercially available devices, which decreases the ability to assess microvascular complications of retinal vascular diseases. In addition, OCTA requires multiple acquisitions in the same anatomic location, limiting the ability to acquire interpretable images in eyes with unstable visual fixation and motion artifacts from microsaccades^5^.

Deep learning, a relatively new subfield of artificial intelligence (AI)^6^, has already played a transformative role in the field of ophthalmology^7,8^. Automated disease classifiers based on ocular imaging are poised to disrupt the traditional delivery of care models by providing status of disease at the point of care^9–14^. In addition, end-to-end feature segmentation provide rich quantification and interpretation of many ocular features^15–17^. Traditionally these supervised machine learning techniques require a large number of expert-defined labels, with two main limitations^18^. First, most of these labels are manually generated by clinicians, which is a cumbersome, time-consuming, and consequently costly process. Using the human generated annotations as the ground truth limits the learning ability of the AI, given that it is problematic for AI to surpass the accuracy of humans, by definition. In addition, expert-generated labels suffer from inherent inter-rater variability, thereby limiting the accuracy of the AI to at most variable human discriminative abilities. Thus, the use of more accurate, objectively-generated annotations would be a key advance in machine learning algorithms in diverse areas of medicine.

Given the relationship of OCT and OCTA, we sought to explore the deep learning’s ability to first infer between structure and retinal vascular function, then generate an OCTA-like en-face image from structural OCT image alone. By taking OCT as input and using the more cumbersome, expensive modality, OCTA, as an objective training target, deep learning could overcome limitations with the second modality and circumvent the need for generating labels.

A successful model would result in the acquisition of new information from preexisting databases given the ubiquitous use of OCT and may result in en-face images significantly less affected by artifacts. Unlike current AI models which are primarily targeted towards classification or segmentation of images, to our knowledge, this is the first application of artificial neural networks in ophthalmic imaging to generate a new image based on a different imaging modality data. In addition, this is the first example in medical imaging, to our knowledge, where expert annotations for training deep learning models are bypassed by using objective, functional flow measurements.

## Results

**Figure 1:**
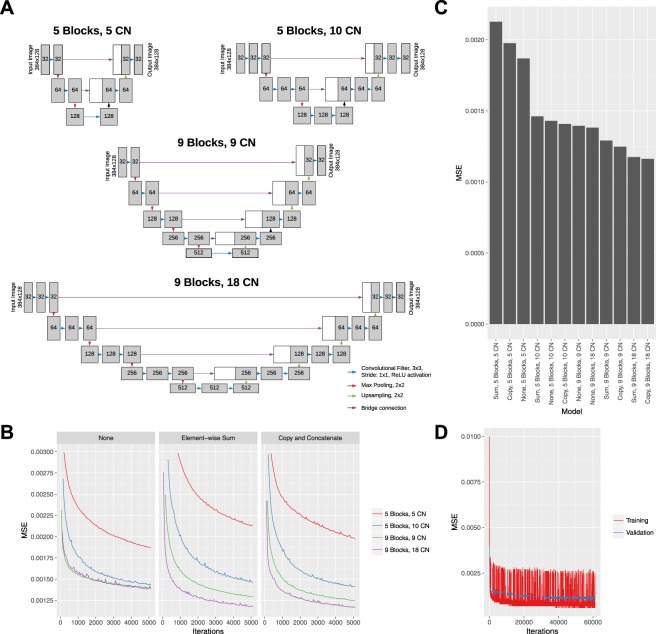
Schematics of deep learning model architectures tested by varying block depth and number of convolutional (CN) filters (**A**). Learning curve for all models with Mean Standard Error (MSE) for validation sets with the same batch size and learning rate and faceted by bridge type (**B**). Lowest MSE after 5,000 iterations for all models (**C**). Learning curves for the best performing (9 blocks, 18 convolutional filters, copy and concatenation bridge) on training and validation set for an extended training session (**D**).

Four different model archetypes (Fig. 1A) were designed to take a single individual structural B scan image as input and provide an inferred flow B scan image as output which included 5 blocks of max pooling and upsampling with 5 convolutional filters, 5 blocks with 10 convolutional filters, 9 blocks with 9 convolutional filters, and 9 blocks with 18 convolutional filters. In addition for each set of the 4 models, 3 different bridge connections were tested: no bridge (similar to traditional convolutional autoencoder network), element-wise summation, and copy + concatenation. Each of these models were trained from random initialization with the same batch number, training/validation datasets, optimizer, and learning rate for 5,000 iterations (Fig. 1B) and the deepest model with 18 convolution filters and with copy + concatenation bridges had the lowest MSE (Fig. 1C). The final model had a total of 7.85 million trainable parameters, has a space complexity of 90 megabytes, and took 6 days of training time. The model received no additional information regarding the neighboring slices. An extended training session was performed with dropout layers for regularization (Fig. 1D). After collecting independently inferred flow B scan images, an en-face projection image was created using the same techniques as OCTA.

A total of 401,098 cross-sectional structural, macular spectral-domain (SD) OCT images from 873 volumes were designated as a training set: these were presented to the deep learning model, and the output was compared to the corresponding retina-segmented OCTA image. Another 76,928 OCT images from independent 171 cubes were used for validation against OCTA images. A held out test set of 92,606 images of 202 cubes from a different set of patients was then used for comparison of deep learning model performance against the OCTA images.

**Figure 2:**
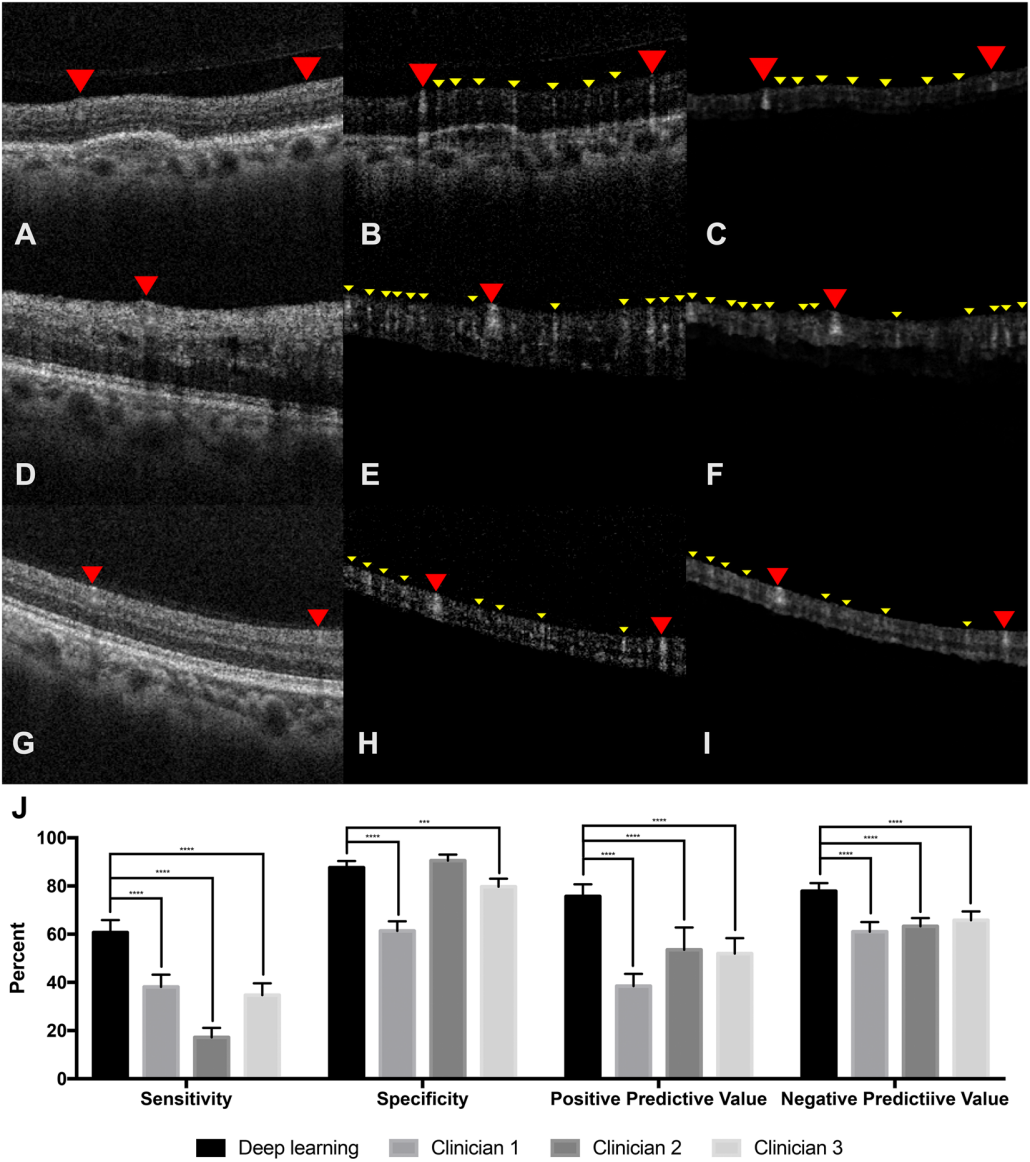
(**A**,**D**,**G**) Example structural OCT images from held-out test set which serves as input for the deep learning model. The red arrowheads point to a large retinal vessel that is easily identified by expert clinicians. (**B**,**E**,**H**) Example cross-sectional OCTA images from held-out test set. The red arrowheads point to the large retinal vessels and the yellow arrowheads point to the small hyperreflective areas that represent the retinal microvasculature and are not apparent on the structural OCT image. (**C**,**F**,**I**) Example cross-sectional images of CNN output from held-out test set identifying retinal vessels. The deep learning model identifies both the large retinal vessels (red arrowheads) and small (yellow arrowheads) microvasculature (yellow arrowheads) similar to the OCTA images. (**J**) Comparison of deep learning model against three masked retina-trained clinicians using OCTA as reference. ***P < 0.0001, ****P < 0.00001.

The model was trained with 60,000 iterations. The learning curve with the mean squared error (MSE) of the training iterations and the validation set are shown in Fig. 1B. The model achieved a minimal validation MSE of 9.9482 × 10^−4^. The weights that produced the best validation MSE were then used for comparison against OCTA. The performance on the held-out test set achieved an MSE of 7.7665 × 10^−4^. The fidelity by peak signal to noise ratio was 31.10 db. Figure 2 shows examples of deep learning inference of retinal flow from cross-sectional structural OCT images of the held out test set compared to the corresponding OCTA images. From structural OCT images, the deep learning model was able to identify both the large and medium-sized retinal vessels as well as the retinal microvasculature at a level of detail similar to the OCTA image. In addition, the model identified small vessels that are not apparent on the structural OCT image. Furthermore, the model was able to learn the segmentation of the retina and isolate structural features of the retina. Given that the model takes as input only a single structural B scan image, we asked three masked independent retina specialty trained experts (CSL, AT, PM) to identify vessels on four structural B scans image from four different volumes. Comparison of model output to three different masked clinicians revealed that the trained model was able to significantly outperform clinicians in terms of specificity, positive predictive value, and negative predictive value when using OCTA as ground truth.

**Figure 3:**
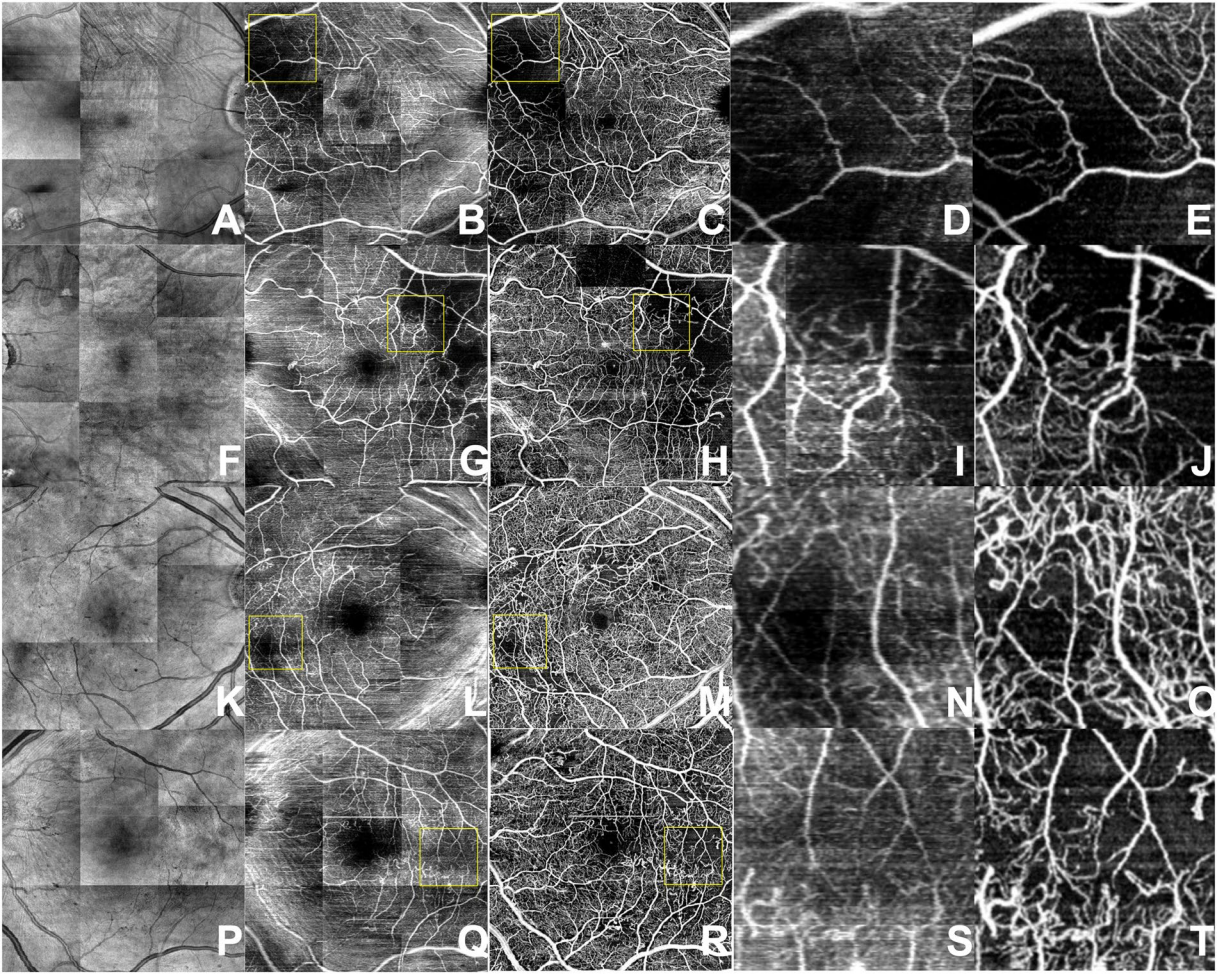
Diabetic retinopathy. En-face projection maps of retinal blood flow created from structural OCT volumes (**A,F,K,P**), AI-generated inferred flow volumes (**B,G,L,Q**) and OCTA flow volumes (**C,H,M,R**). Magnified views of the AI-generated (**D,I,N,S**) and OCTA (**E,J,O,T**) images where decreased blood flow is contrasted with normal flow. Deep learning images demonstrate similar details of superficial retinal vasculature as the OCTA image while missing higher density of deep capillary networks which are visible on OCTA images.

**Figure 4:**
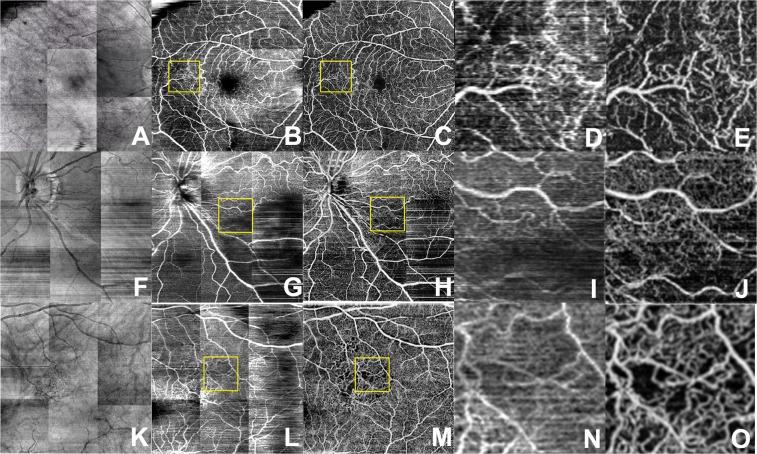
Branch retinal vein occlusion. En-face projection maps of retinal blood flow created from structural OCT volumes (**A,F,K**), AI-generated inferred flow volumes (**B,G,L**) and OCTA flow volumes (**C,H,M**). Magnified views of the AI-generated (**D,I,N,S**) and OCTA (**E,J,O,T**) images where the decreased blood flow is contrasted with normal flow.

**Figure 5:**
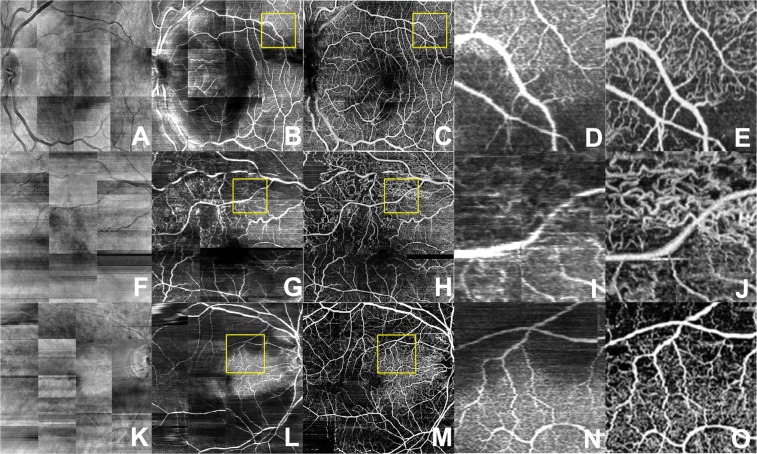
Central retinal artery occlusion. En-face projection maps of retinal blood flow created from structural OCT volumes (**A,F,K**), AI-generated inferred flow volumes (**B,G,L**) and OCTA flow volumes (**C,H,M**). Magnified views of the AI-generated (**D**,**I**,**N**) and OCTA (**E**,**J**,**O**) images where decreased blood flow is contrasted with normal flow.

To examine the performance of the model on other retinal pathologies outside of what the algorithm was trained, the weights from the lowest validation MSE were used to infer flow of each cross-sectional structural OCT image in a volume. Average projection of resulting inferred flow volume was used to create en-face projection maps of flow (Figs 3–5). Surprisingly, even without three-dimensional knowledge of the location of vessels nor knowledge of the neighboring cross sectional slice, the inferred flow by deep learning generated contiguous vessel maps similar to OCTA.

Compared to en-face projections of the structural OCT volumes, the AI generated flow maps showed more detail of the superficial retinal vasculature (Figs 3–5). The map of retinal vasculature generated by the model was much more detailed at the superficial retina than deeper in the retina and was superior to structural OCT en-face projections. This discrepancy was easily demonstrated in the pathologic eyes (Figs 3–5; Supplementary Figs 1–3) in which the superficial capillary plexus were affected by ischemia more than deep capillary plexus. In eyes with diabetic ischemia (Fig. 3), branch retinal vascular occlusion (Fig. 4), and cilioretinal artery occlusion (Fig. 5), the AI generated flow maps were superior to en-face projections of the structural OCT volumes and similar to OCTA in showing the disrupted flow within the superficial retinal vasculature in various pathologies (Supplementary Figs 1–3). As expected, OCTA was superior in showing deeper retinal vasculature than the model’s flow output and revealed a higher density of deep capillary plexus in these diseases (Supplementary Figs 1–3). Intact cilioretinal artery in the setting of central retinal artery occlusion (CRAO) preserves the superficial capillary plexus in the area perfused by cilioretinal artery. As expected, our model’s results were comparable to OCTA in the area perfused by cilioretinal artery but not able to show the remaining deep capillary plexus elsewhere in the macula (Fig. 5L–O).

**Figure 6:**
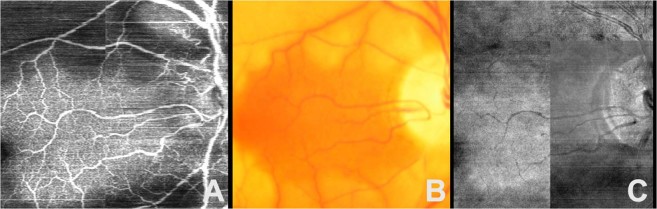
En-face projection maps of retinal flow created from inferred flow by deep learning (**A**) compared to the corresponding color fundus photo (**B**) and structural OCT en-face projection (**C**) of an eye with central retinal artery occlusion and intact cilioretinal artery.

**Figure 7:**
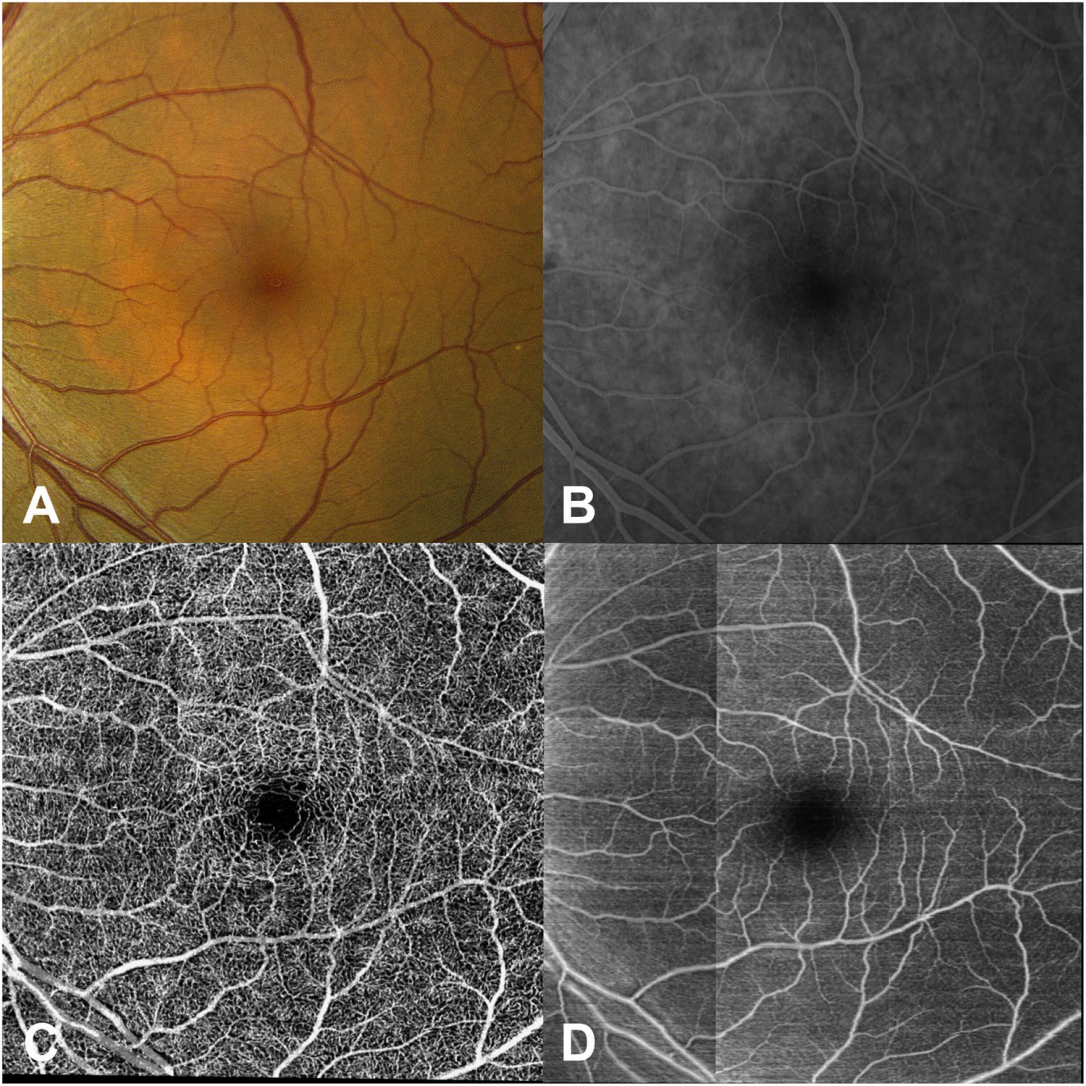
Color fundus photo (**A**) of a normal retina compared to the corresponding late phase fluorescein angiogram (**B**), the corresponding OCTA image (**C**), and contiguous vessel map generated by the deep learning model (**D**).

**Figure 8:**
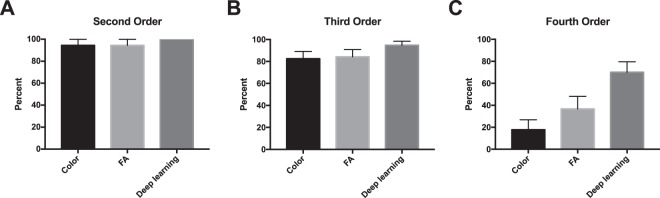
Manual segmentation of vessels on color, fluorescein angiography (FA), and deep learning generated flow maps with OCTA as reference for second order (**A**), third order (**B**), and fourth order vessels (**C**). *P < 0.05, **P < 0.001, ***P < 0.0001.

When compared to color fundus photography and en-face projections of structural OCT volumes, the retinal vasculature map generated by the model was better for visualizing the superficial capillary networks. As shown in Fig. 6, the deep learning image highlights the area of capillary dropout and intact flow. Both superficial arterioles and superficial capillary networks are clearly visible in the area supplied by cilioretinal artery on deep learning image. In contrast, only larger retinal vessels are visible, and the integrities of capillary plexus are difficult to assess on the color photo and structural OCT en-face projections. Similarly, Fig. 7 shows that, for a normal eye, the retinal vasculature map generated by the model demonstrates the superficial capillary networks with superior detail compared to both the corresponding color fundus photograph and the late phase fluorescein angiogram. OCTA continues to highlight the retinal microvasculature with the highest detail. Manual segmentation of vessels on all four imaging modalities was performed (Fig. 8). For second order vessels, 87.5%, 97.5%, and 100% of the vessels were identified by color, FA, and deep learning generated flow maps. Compared to OCTA as the ground truth, the trained model identified fewer third and fourth order vessels. However, deep learning was able to identify significantly more third order vessels compared to color images (p = 0.0320) and was able to identify significantly more fourth order vessels compared to both color and FA images (p = 1.86 × 10^−5^ and 5.01 × 10^−4^ respectively). Finally, the trained model was successfully able to generalize to a different OCT imaging device and predict the areas of non-perfusion in a patient with branch retinal vein occlusion (Supplementary Fig. 5).

## Discussion

Our study demonstrates that a deep learning model can be trained to recognize features of OCT images that allow successful identification of retinal vasculature on cross sectional OCT scans in a fully automated fashion and generate en-face projection flow maps. The deep learning model identified both the retinal vessels that were easily seen on structural OCT B-scan images as well as the retinal microvasculature that was not apparent to the clinician on standard OCT and showed significantly more retinal vessels compared to structural OCT en-face projections, color and FA images. Surprisingly, deep learning was able to generate detailed flow maps of the retinal vessels in a variety of retinal conditions using standard, ubiquitously available structural imaging.

Taking advantage of an already existing imaging modality as the ground truth, our study bypasses the need to generate expert annotations entirely. Furthermore, acquiring a retinal vascular map from OCT via deep learning enables the comparison of retinal vascular structure versus function of retinal vasculature using OCT and OCTA, respectively. One of the potential explanations by which deep learning infers flow from static images relate to speckle formation from the optical system^19,20^. The OCT uses coherent light source, therefore, speckle formation is inevitable from the optical interference due to heterogeneous properties of biological tissue. In fact, OCT imaging system is a combination of numerous laser speckle imaging systems. If the tissue target is static, then the speckle pattern in the OCT image is fixed and determined by optical system setup (numerical aperture, wavelength used, etc) and the size of scatters. However, when there is localized blood flow within tissue, the speckle pattern at that localized position will tend to become finer. The faster the speed of the flow, the finer the speckle pattern would appear in the static optical image that is captured. Therefore, the statistical properties of localized spatial speckle pattern (for example variance) would be a good surrogate to infer the dynamic information from the static optical images. In fact, this phenomenon is widely used in the spatial laser speckle imaging to infer dynamic blood perfusion within living tissue. Speckle variance imaging measures decorrelation between the OCT signals that are generated by speckle or backscattered light from biological tissues, albeit without the ability that our model is capable of, i.e. to delineate the dynamic blood flow maps of the scanned tissue volume^19,20^.

To illustrate that our model is generating functional vascular map from structural, static images, we blurred the structural OCT images that are used as input for the model. As the structural images were blurred, the AI output became progressively more different from the OCTA images, indicating that the model is likely decoding subtle functional information found in structural images (Supplementary Fig. 4).

The novel application of deep learning in our study infers flow from traditional OCT images. This finding has significant clinical applications. OCT is the most commonly performed eye procedure, thus resulting in extensive OCT databases in most clinics (including those acquired before OCTA was available). This large imaging cohort may allow us to determine the natural history of vascular changes, blood flow, and clinical outcomes in retinal diseases, similar to previous studies involving fundus photographs^21,22^ but allowing much precise 3D volumetric information of retinal vasculature and thickness of retinal layers. These algorithms may also enhance the image quality of OCTA machines and reduce the number of repeated frames that need to be captured. Finally, future comparisons of the OCTA and deep learning images of the eyes that underwent a recent vascular insult, in which a clear difference exists between structure and retinal blood flow would be useful.

Neural networks have been used to translate between two structural imaging modalities such as from MRI to CT^23–27^. Rather than translating between structural images, our AI algorithm was able to generate functional flow images from structural images, which may have further clinical applications. More research is needed to establish the utility of applying deep learning to structurally correlated images, but a similar principle could be applied in different fields of medicine where structural imaging is routinely obtained and functional imaging data is available for use of the ground truth, such as computerized tomography or magnetic resonance imaging, and angiography images may be generated.

In comparison to structural OCT en-face projections, we show that the deep learning inferred flow maps are able to provide better definition of retinal vessels (Figs 3–6). Powner *et al*. has previously shown that en-face projections of the structural OCT volumes leave behind basement membranes which are unperfused but will appear hyperreflective on the structural OCT imaging^28^. This suggests that the AI generated flow maps may have more clinical utility than en-face projections of structural OCT volumes since the latter cannot distinguish between perfused and unperfused vessels. We also demonstrate that the AI algorithm was able to generate a flow map to show the preservation of the flow in the intact cilioretinal artery in the case of central retinal artery occlusion (Figs 5 and 6), where the majority of the retinal blood flow in the macula is acutely lost.

In our work, we utilized a U shaped autoencoder network as the final model that has traditionally been used for semantic medical segmentation^29^. This deep neural network architecture uses bridges to maintain high resolution spatial information that is normally lost during pooling operations. In addition, deeper neural networks have generally been found to improve performance which has been shown in the computer vision research with improved accuracy with ImageNet image classification as networks became deeper^30–32^. With our data, we have empirically found that the copy and concatenation bridge with deeper number of convolutional layers led to the best performance compared to models with shallower networks, no bridge connections, and element-wise summation bridge connections. Future work could include further optimization and hyperparameter model selection and comparison to other architectures such as V-net^33^, recurrent convolutional layers^34^, two-pathway convolutional networks^35^, 3D convolutional models, and hybrid models with convolutional layers with recurrent network layers. In addition, future analyses should include evaluation of accuracy of synthetic flow reconstruction in different sublayers of retina using either automated or manual retinal sublayer segmentation.

More broadly, a similar approach could have application in many other imaging modalities, where the same object is imaged with sensitivity to different properties of the object that is being imaged. For example, radiological/MRI measurements of the same body part are routinely conducted with different contrasts, taking advantage of the sensitivity of different contrasts to different properties of the tissue. While this allows measurements of different tissue properties, it is also time-consuming. In some cases, measurements that are highly sensitive to certain tissue properties may require invasive injection of contrast agents, or exposure to x-ray radiation. The present results demonstrate that even a very small amount of sensitivity to variations in a tissue property may be enough for a deep neural network algorithm to detect variations in the dependent image properties, enabling accurate inference of the physiological features of the imaged body part, even when these features are not readily visible to an expert, and undetectable by means of other image processing algorithms. For example, MR angiography (MRA) and anatomical MRI are often both required for imaging of soft tissue and imaging of blood vessels in the same organ. If the anatomical MRI possesses subtle sensitivity to the structure of the blood vessels, as demonstrated in the present study for OCT, it is possible that information analogous that derived from MRA could be inferred directly from an anatomical MRI scan.

Our approach has a number of limitations. The study data was collected at a single academic center with a device from a single manufacturer and a consistent imaging protocol. While this may limit the immediate generalizability of this method, the weights learned in this work could be used as the starting point for transfer learning^36,37^, allowing the model to learn to infer with images from other devices rapidly, and with substantially less data. Indeed, we have shown that without retraining the model is able to recapitulate the areas of capillary dropout with a different OCT device (Supplementary Fig. 5). Future studies will need to evaluate the clinical correlations between our deep learning inferred flow maps and retinal perfusion which would include oxygenation of vascularized tissue, tissue oxygen consumption, and/or flow velocity. Lastly, our deep learning approach used images from diabetic patients as the training set since we believed that diabetic manifestations such as microaneurysms, hard exudates, macular edema, neovascularization, and capillary non-perfusion encompass the majority of the OCTA features present in other retinal vascular diseases. While this strategy may limit the generalizability of the model to other retinal vascular diseases, the results from the test set demonstrate that model was not overfitting on diabetic specific features. Ultimately collecting a comprehensive, balanced training set with the universe of all possible retinal manifestations may be insurmountable, and it is encouraging that our model was able to generalize when only trained on a subset of disease features.

In conclusion, we show that deep learning is able to generate flow maps of superficial retinal circulation using structural OCT images alone. This approach may be used to analyze existing OCT datasets or be integrated into existing OCT machines today. In addition, this methodology of inferring weakly correlated images may be useful in many other imaging applications.

## Methods

Patients with any retinal diagnoses seen in retina clinics at the University of Washington, Seattle, WA were included in the study. Patients were imaged with both Spectralis OCT (Heidelberg Engineering, Heidelberg, Germany) as standard of care and OCTA as part of the research protocol. Written informed consent was obtained prior to acquisition of OCTA. Patients younger than 18 or non-English speakers were excluded. OCTA images with significant signal quality problems, including signal strength below 6 or excessive motion artifacts were excluded from the study. This study was approved by the Institutional Review Board of the University of Washington and was in adherence with the tenets of the Declaration of Helsinki and the Health Insurance Portability and Accountability Act. Consecutive cases of diabetic retinopathy, branch retinal vein occlusion, and central retinal artery occlusion were reserved as a held-out test set.

### OCTA imaging

Participants underwent imaging with a 68 kHz CIRRUS™ HD-OCT 5000 with AngioPlex™ OCT Angiography (ZEISS, Dublin, CA), which operates at a central wavelength of 840 nm. To achieve OCTA imaging of retinal vasculature, a repeated B-mode scanning protocol was implemented in the prototype. Four repeated B-scans were acquired at one position and used to extract the blood flow signal as previously described^5^ with the total time for single volume acquisition being 3.6 seconds excluding the adjustment time prior to data collection.

In this study, the OCT Angiography system was equipped with motion tracking through an auxiliary real time line scan ophthalmoscope (LSO) and allowed montaging of images with 245 slices obtained per volume^38^. The minimum array for the grid was 3 × 3, giving a FOV of 6.8 × 6.8 mm^2^ (approximately 30–40 degrees field of view) while maximum array is 4 × 6, providing a coverage of 9.0 × 13.4 mm^2^. Structural OCT volumes as well as the previously described OCTA algorithm^2,39,40^ was applied to all the volumetric datasets, and the large en-face OCTA was obtained by stitching the images and maximum intensity projection by use of a software coded with Matlab^41^. Structural en-face OCT projections were created in the same manner from the structural OCT volumes using average intensity projections. All en-face imaging and evaluation metrics were projected and performed from the whole retina (from the internal limiting membrane to above the retinal pigment epithelium).

### Deep learning

To understand the generalizability of the final deep learning model to other retinal vascular diseases, the dataset was segregated by disease *a priori* and OCTA volumes from diabetic patients were used for training, validation, and test. After training was completed, the model was tested on volumes from other retinal vascular diseases including retinal vein occlusions and retinal artery occlusions.

OCTA and OCT volumes taken from diabetic patients were used for training the deep learning algorithm. We employed a standard deep learning training approach where our dataset was partitioned at the patient level into a training, validation, and held-out test set. Models were trained using the training set and the validation set was used to periodically check the model performance. Model architecture and hyperparameters were selected using the best performance on the validation set. After freezing the weights, the model was then tested on the held-out test set. Hyperparameters such as the learning rate and the batchsize were determined by grid search. 70%/15%/15% of volumetric cubes were randomly assigned to training, validation, and testing, respectively. After applying image registration of the OCTA images to the OCT scans, the retinal layers of the OCTA image were segmented and used as ground truth. The input into the model was the unaltered grayscale structural OCT image of the retina. Both the input and the outputs were scaled between 0 and 1. The images were sliced into non-overlapping, vertical strips of 384 px by 128 px to allow deep learning to access the native resolution of the OCT images. Thus the input tensor size was 384 × 128 in dimension; the height was the native height of the OCT image from the device and the width was empirically determined using grid search. 3D convolutional models were considered but given that OCT imaging devices are not standardized in the spacing between B-scans, we employed 2D convolutional models at the level of B-scan images.

A total of 12 independent deep learning models were tested with varying levels of depth and convolutional filters. Four models were constructed in the style of traditional fully convolutional autoencoder networks with no bridge connection. Four models were constructed with a residual-like element wise summation to combine tensors, and four models were constructed with copy + concatenation to combine tensors in the style of the U-shaped autoencoder^29^ (Fig. 1A). The activation of the final layer was set to linear activation and Mean Square Error (MSE) was used as the loss function during training. The learning rate was set to 1e-5, and the Adam learning function^42^ was used during training. Batch size was set to 400 images, and the loss was recorded at every training iteration. Dropout was used for the extended training session with the best performing model. Validation MSE was calculated at the end of each epoch and training was stopped when the model began to either overfit or the loss function plateaued. All training was performed using a single server equipped with 8 x Nvidia Tesla P100 graphics processing units with Keras (http://github.com/fchollet/keras), Tensorflow (http://www.tensorflow.org), and NVIDA cuda (v8.0) and cu-dnn (v6.0) libraries (http://www.nvidia.com).

To examine the performance of the trained model, structural OCT volumes from non-diabetic patients with other retinal vasculopathies were used as a held out test set. The cross-sectional structural OCT images from each retina was passed independently through the trained model for inference and projection was performed on the resulting volume. The en-face images from the deep learning output were then compared to the en-face images generated from OCTA.

Comparison against clinicians of ability to identify vessels on structural B scan images was performed by sending four full B scan structural images (384 × 256 pixels) from four different volumes and having three masked retina trained experts segment on the images, utilizing any image enhancement softwares as necessary. Four images were chosen as the grading task generates 393,216 data points for paired comparison at the pixel level, and sufficient statistical power could be achieved. The OCTA B scan image was used as ground truth, and the model output was used to compare using paired study design. Each of the retinal experts provided binary segmentation masks for the OCT pixels they believed would be predictive of flow on the corresponding OCTA B-scan. The deep learning prediction and the OCTA flow B scan was also binarized and the metrics (sensitivity, specificity, positive predictive value, and negative predictive value) were calculated per pixel. Sensitivities and specificities were compared using the McNemar test and the positive and negative predictive values were compared using a generalized score statistic^43^.

Comparison of the model output en-face image against OCTA en- face image, color imaging and fluorescein angiography was performed by registering the four modalities to each other and then randomly sampling 100 × 100 pixel squares and asking a retina trained clinical expert to segment and label each vessel as second, third, or fourth order vessels. OCTA was used as the reference and the percent visible was calculated for each modality. Fisher exact test was used to calculate significance.

To assess the ability of the model to generalize to a different OCT device, OCT and OCTA images were obtained from the Topcon Triton OCT device. The structural OCT images were normalized by histogram equalization and then used as input into the model without retraining. The resulting flow B-scans were then projected using average intensity projections to create the en-face maps.

## Supplementary information

Supplementary figures

## Data Availability

The datasets generated during and/or analyzed during the current study are not publicly available due to patient privacy and violation of informed consent.
