# Supplementary material for: Generating retinal flow maps from structural optical coherence tomography with artificial intelligence
Source: Sci Rep. 2019 Apr 5;9:5694. doi: 10.1038/s41598-019-42042-y (PMC6450899; doi:10.1038/s41598-019-42042-y)
Supplement: Supplementary file 1 — Supplementary figures [file 41598_2019_42042_MOESM1_ESM.pdf]

Generating retinal flow maps from structural optical coherence tomography with artificial intelligence

Cecilia S. Lee MD MS,[1] Ariel J. Tyring MD,[1] Yue Wu PhD,[1] Sa Xiao PhD,[1] Ariel S. Rokem PhD,[2] Nicolaas P. Deruyter BS,[1] Qinqin Zhang PhD,[3] Adnan Tufail MD FRCOphth,[4] Ruikang K. Wang PhD,[1,3] Aaron Y. Lee MD MSCI*[1,2]

[1] Department of Ophthalmology, University of Washington, Seattle WA

[2] eScience Institute, University of Washington, Seattle WA

[3] Department of Bioengineering, University of Washington, Seattle WA

[4] Moorfields Eye Hospital NHS Foundation Trust, London UK

Corresponding author:

Aaron Y. Lee
Assistant Professor
Department of Ophthalmology
University of Washington
Box 359608, 325 Ninth Avenue
Seattle WA 98104
Ph: (206) 543-7250
Email: leeay@uw.edu

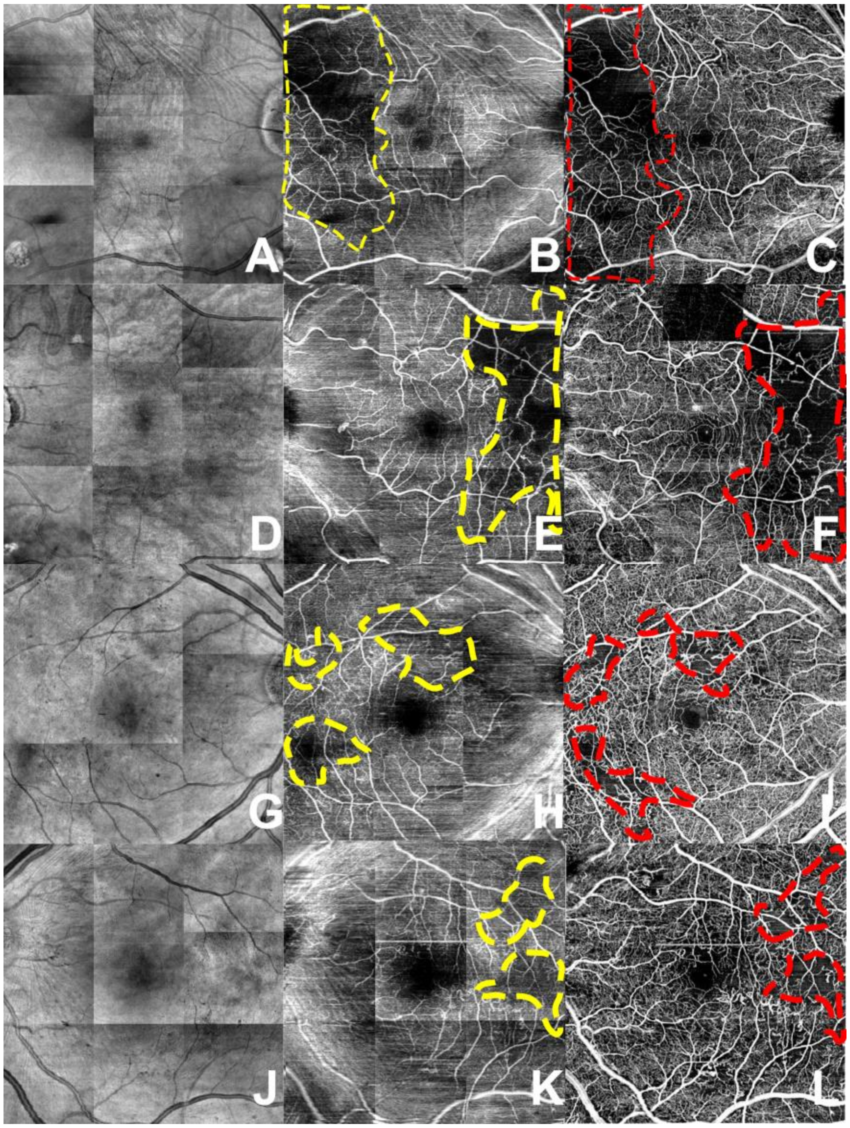

Supplementary Figure 1: Retinal vascular areas without flow in diabetic retinopathy. Panels A, D, G, J show en-face structural OCT projection images. Panels B, E, H, K show en-face projection of deep learning inference of flow with yellow dotted lines highlighting areas without flow. Panels C, F, I, L show en-face projection of OCTA with red dotted lines highlighting areas without flow.

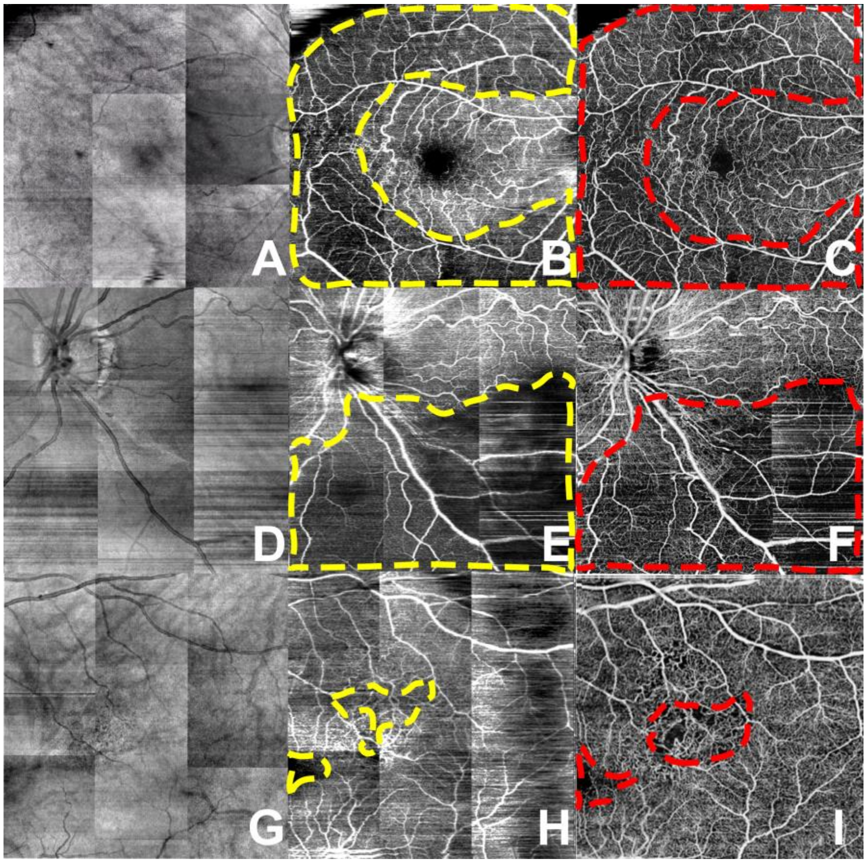

Supplementary Figure 2: Retinal vascular areas without flow in retinal vein occlusion. Panels A, D, G show en-face structural OCT projection images. Panels B, E, H show en-face projection of deep learning inference of flow with yellow dotted lines highlighting areas without flow. Panels C, F, I show en-face projection of OCTA with red dotted lines highlighting areas without flow.

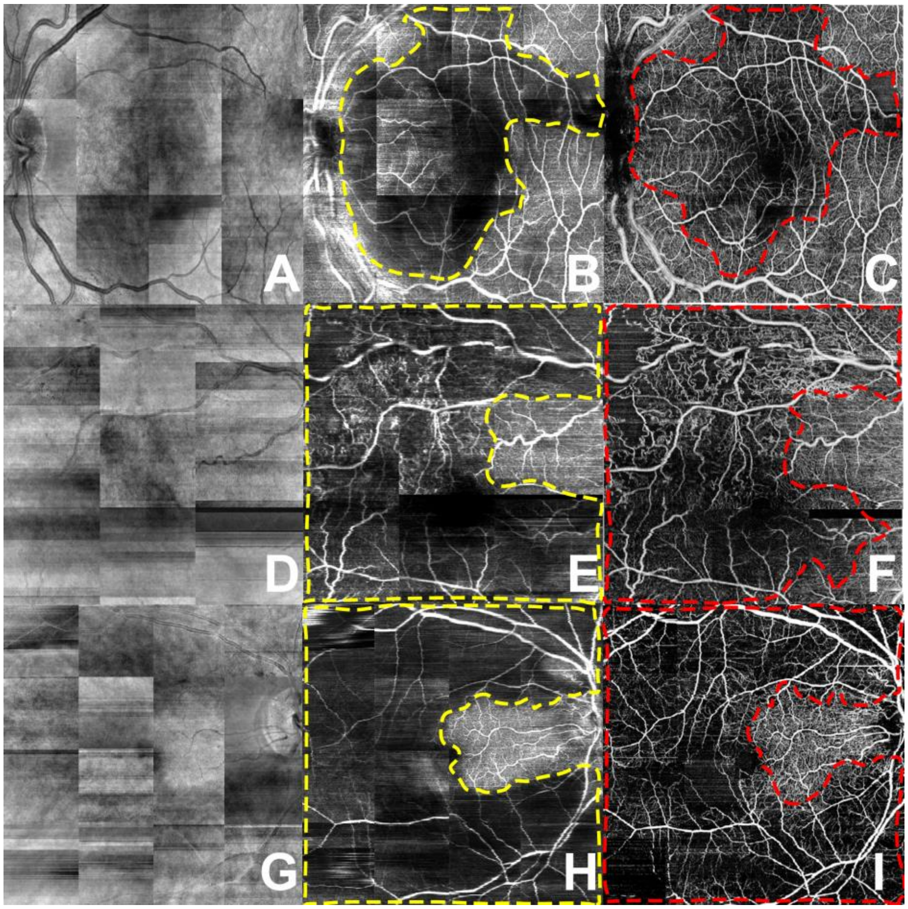

Supplementary Figure 3: Retinal vascular areas without flow in central retinal artery occlusion. Panels A, D, G show en-face structural OCT projection images. Panels B, E, H show en-face projection of deep learning inference of flow with yellow dotted lines highlighting areas without flow. Panels C, F, I show en-face projection of OCTA with red dotted lines highlighting areas without flow.

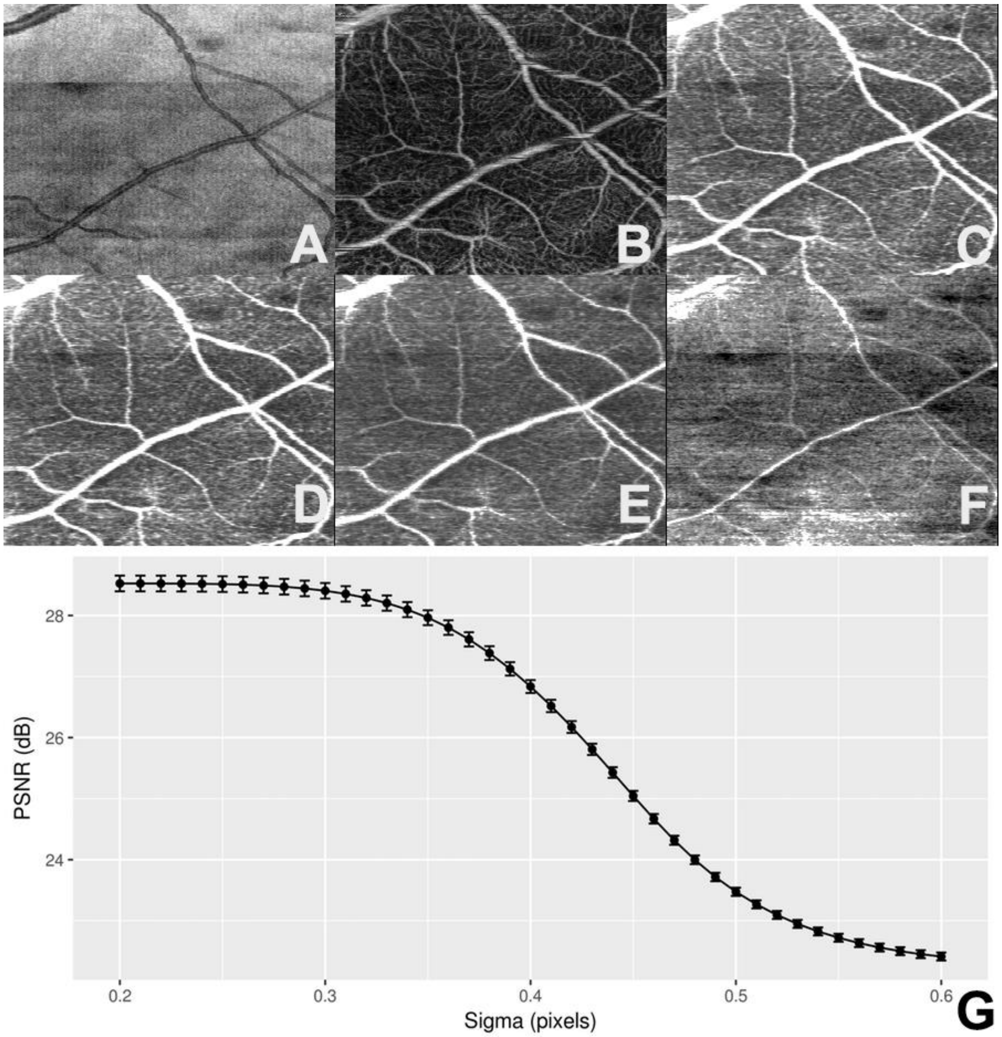

Supplementary Figure 4: Progressive Gaussian blurring of input OCTs. Structural OCT, OCTA, and Deep-learning inferred flow en-face projections are shown in panel A, B, and C respectively. With 2D Gaussian blurring, sigma was varied to create more and more blur and a corresponding drop in the Peak Signal-to-Noise Ratio (PSNR) was noted compared to the OCTA image (Panel E). Panels D, E, F show the output of the model of blurred input with sigma set at 0.2, 0.4, and 0.6 pixels, respectively, indicating that information for inference of smaller vessels are encoded as high spatial resolution information.

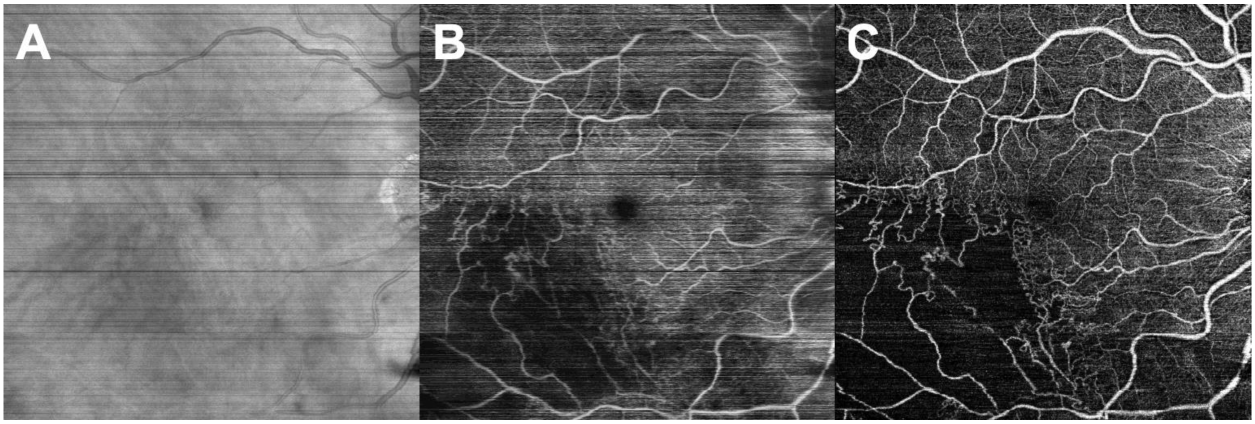

Supplementary Figure 5: Generalization results for Topcon OCT device. Structural OCT, Deep-learning inferred, and OCTA flow en-face projections are shown in panel A, B, and C respectively in a patient with branch retinal vein occlusion without retraining. The previously trained deep learning model is able to accurately reconstruct areas of non-perfusion from the structural OCT B scan images.
